# Pancreatic cancer outcome—local treatment with radiation using MRI-LINAC

**DOI:** 10.3389/fonc.2023.1289919

**Published:** 2023-11-24

**Authors:** Galit Almog, Raphael M. Pfeffer, Svetlana Zalmanov, Vladislav Grinberg, Yoav Lipsky, Elena Chernomordikov, Daphne Levin, Sara Apter, Orit Arsenault, Dan Epstein, Qusai Tamimi, Keren Hod, Dror Limon, Talia Golan, Irit Ben-Aharon, Yaacov Richard Lawrence, Merav Akiva Ben-David

**Affiliations:** ^1^ Goldman School of Medicine, Ben Gurion University of the Negev, Beer Sheva, Israel; ^2^ Radiation Oncology Department, Assuta Medical Center, Tel Aviv, Israel; ^3^ Faculty of Health Sciences, Ben Gurion University of the Negev, Beer Sheva, Israel; ^4^ School of Medicine, Tel Aviv University, Tel Aviv, Israel; ^5^ Department of Academy and Research, Assuta Medical Center, Tel Aviv, Israel; ^6^ Radiation Oncology Department, Rabin Medical Center, Petah-Tikva, Israel; ^7^ Radiation Oncology Department, Sheba Medical Center, Ramat-Gan, Israel; ^8^ Oncology Department, Rambam Medical Center, Haifa, Israel

**Keywords:** SMART, MRgRT, pancreatic cancer, re-irradiation, radiation toxicity

## Abstract

**Introduction:**

Stereotactic MR-guided on-table adaptive radiotherapy (SMART) allows the precise delivery of high-dose radiation to tumors in great proximity to radiation-sensitive organs. The aim of this study is to evaluate the toxicity and clinical outcome in locally advanced or recurrent pancreatic tumors, with or without prior irradiation, treated with SMART.

**Methods:**

Patients were treated for pancreatic cancer (PC) using SMART technology to a prescribed dose of 50 Gy (BED_10_, 100 Gy) in five fractions, with daily on-table adaptation of treatment plan. Endpoints were acute and late toxicities, local control, local disease-free period, and overall survival.

**Results:**

A total of 54 PC patients were treated between August 2019 and September 2022, with a median follow-up of 8.9 months from SMART. The median age was 70.4 (45.2–86.9) years. A total of 40 patients had upfront inoperable PC (55% were locally advanced and 45% metastatic), and 14 had local recurrence following prior pancreatectomy (six patients also had prior adjuvant RT). Of the patients, 87% received at least one chemotherapy regimen (Oxaliplatin based, 72.2%), and 25.9% received ≥2 regimens. Except from lower CA 19-9 serum level at the time of diagnosis and 6 weeks prior to SMART in previously operated patients, there were no significant differences in baseline parameters between prior pancreatectomy and the inoperable group. On-table adaptive replanning was performed for 100% of the fractions. No patient reported grade ≥2 acute GI toxicity. All previously irradiated patients reported only low-grade toxicities during RT. A total of 48 patients (88.9%) were available for evaluation. Complete local control was achieved in 21.7% (10 patients) for a median of 9 months (2.8–28.8); three had later local progression. Eight patients had regional or marginal recurrence. Six- and 12-month OS were 75.0% and 52.1%, respectively. Apart from mild diarrhea 1–3 months after SMART and general fatigue, there were no significant differences in toxicity and outcomes between post-pancreatectomy and inoperable groups.

**Conclusion:**

SMART allows safe delivery of an ablative dose of radiotherapy, with minimal treatment-related toxicity, even in previously resected or irradiated patients. In this real-world cohort, local control with complete response was achieved by 20% of the patients. Further studies are needed to evaluate long-term outcome and late toxicity.

## 1 Introduction

Exocrine pancreatic cancer (PC) is a common and highly lethal malignancy. It is the fourth and seventh leading cause of cancer-related death in the US and worldwide, respectively, with a 5-year overall survival (OS) rate of 12.5% (1) Most pancreatic cancer patients succumb to distant metastatic disease. Patients without evidence of metastases at diagnosis are considered for surgical resection, but most of these tumors are considered inoperable due to tumor involvement of regional blood vessels. Patients with locally inoperable disease often receive chemotherapy in an attempt to shrink the tumor and convert it to resectable, yet only 15%–20% of patients are operable (2) and prognosis is poor even after complete resection, due to frequent metastatic disease and high rates of both systemic and local recurrence (3, 4) Conventional radiotherapy has been studied in these non-resectable patients and has not been found to contribute to long-term local control or survival. On the other hand, dose-escalated radiotherapy delivered in 15–25 fractions to a biologically effective dose (BED) of 98 Gy has been shown to have good local control (local failure 17.6% at 1 year and 32.8% at 2 years) and moderate survival (38% at 2 years) compared to historical control (5).

Stereotactic body radiotherapy (SBRT) is often given as salvage treatment for local inoperable or locally recurrent PC progressing after chemotherapy (6) SBRT delivered to pancreatic cancer without online image guidance is limited to doses of approximately 35 Gy in five fractions due to the risk of toxicity to adjacent critical organs such as the stomach and duodenum. Retrospective studies demonstrated optimistic local control outcomes but no change in OS and provoked some concerns regarding treatment toxicities (7) The use of CT imaging (on-board cone beam CT) in abdominal RT provides limited soft tissue contrast and inability to perform real-time tracking to account for internal organ movement (8). In practice, this translates to subtherapeutic doses of RT in an effort to reduce OAR toxicities (9–11). On the other hand, safely delivering higher doses of radiation may improve long-term local control (LC) and OS. A prospective randomized study showed no benefit from such doses of SBRT following systemic chemotherapy (12).

Stereotactic MR-guided adaptive radiotherapy (SMART) allows delivery of ablative dose to abdomino-pelvic tumors, even when adjacent to OARs. This is possible due to continuous real-time MR-based imaging of internal structures with improved soft tissue visualization compared with CT (8), daily on-table adaptive replanning, and automatic beam delivery cessation based on real-time target position tracking (13, 14). Recent studies have shown that SMART is safe, allows dose escalation with OAR sparing (15), and may improve OS in patients with inoperable PC (16). The development of real-time imaging with MRI allows safe delivery of higher doses of hypofractionated SBRT while ensuring that the dose to the adjacent organs at risk is limited to below what is considered a toxic dose. The recently completed multi-institutional SMART study showed that a dose of 50 Gy in five fractions (BED = >100 Gy_10_ can be delivered with no grade 3 toxicity (17).

While recent studies have shown promising results, research regarding the effectiveness of SMART in PC treatment has mainly focused on primary inoperable cases, leaving little evaluation of cases with local recurrence of PC after surgery or previously irradiated patients (18). This study seeks to evaluate the outcomes and toxicity of SMART in treating primary inoperable, post-surgery locally recurrent, and previously irradiated PC patients. This is the first study, to the best of our knowledge, to assess and compare the effectiveness of SMART in patients with post-operative or recurrent PC.

## 2 Methods

### 2.1 Study population

This is a retrospective cohort study enrolling 54 consecutive patients with pancreatic malignancy treated with SMART using MRI-LINAC system (ViewRay Inc. MRIdian ^®^, Oakwood Village, OH, USA) between August 2019 and September 2022. Patients with inoperable (unresectable/metastatic), medically inoperable (poor performance status, multiple associated morbidities), or recurrent PC following pancreatectomy were included, and patients with either prior pancreas-directed radiation or evidence of distant metastases were also included in this study. All patients signed informed consent, and the study was approved by the institutional IRB committee (ASMC-0078-22).

### 2.2 Treatment

Patients underwent MRI simulation using the MRI-LINAC (supine position, both arms above the head, and 400 cc water PO 45 min prior to scanning) followed immediately by CT-based simulation in the same position.

For treatment planning, gross tumor volume (GTV) and OARs (stomach, duodenum, small and large bowel, kidneys, aorta, inferior vena cava, and spinal cord) were contoured on the MR simulation imaging after fusion with pre-treatment imaging (MRI and/or PET-CT). All contours were reviewed by an expert radiologist (SA) prior to planning. Planning target volume (PTV) was generated from GTV with a 3-mm margin. Our planning risk volume (PRV), which was also the optimization structure, was generated by cropping the PTV from OARs with an additional 3 mm to allow for dose fall-off.

**Figure 1:**
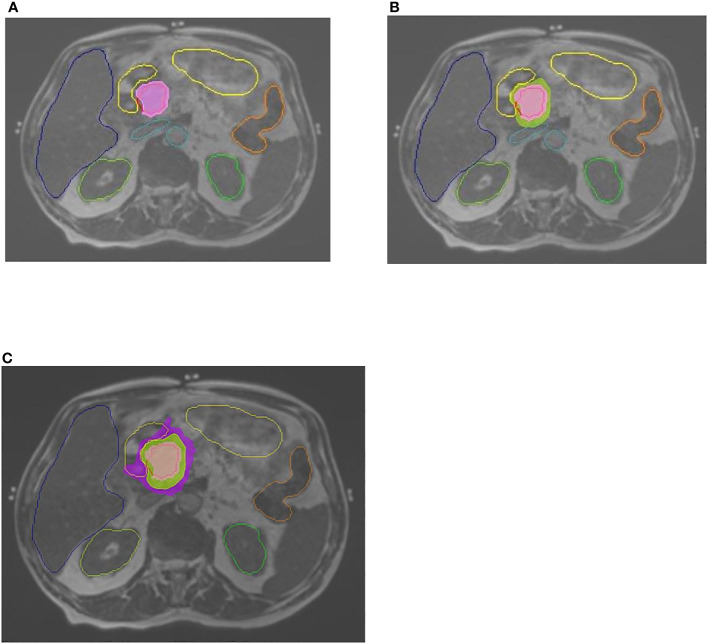
Contouring and doses. Abdominal MRI, 0.35T, with contoured OARs: stomach-duodenum, liver, kidneys, large bowel, great vessels. **(A)** GTV contoured in red. Pink color wash–100% dose (of 50 Gy prescribed dose). **(B)** Green color wash—95% dose. **(C)** Purple color wash—50% dose.

The prescription dose was 50 Gy in five fractions (BED_10_, 100 Gy) delivered on alternate days, with the goal of 95% PRV coverage with 95% of prescribed dose (47.5 Gy). Dose limits for OAR were as follows: for the duodenum, stomach, and small and large bowels, the maximum dose constraint was V33^^1^^ ≤ 0.5 cm^3^. The goal for the liver was to achieve a mean dose of <20 Gy while keeping 700 cm^3^ under 15 Gy. For the spinal canal, the constraint was a V25^^2^^ ≤ 0.5 cm^3^. The constraint for each kidney was mean dose of <12 Gy, with no more than two-thirds of each kidney receiving a dose higher than 14 Gy. If one of these structures exceeded the dose–volume constraint, treatment plan was adapted accordingly to adhere to dose–volume constraints. See **Figure 1** for contouring and doses. The treatment was delivered using equally spaced 19–23 fields, with 50–65 segments, and filter-free 6 MV beam energy with 600 mu/minute dose rate. The Monte Carlo calculation algorithm was used (proprietary ViewRay algorithm).

Three patients received concomitant SBRT to celiac lymph nodes to a dose of 35 Gy in five fractions, and five patients received concomitant SBRT to a liver metastasis (50 Gy in five fractions). Patients did not receive chemotherapy during radiation treatment period.

On-board MRI was performed prior to each fraction, and the OARs and the GTV, PTV, and PTV_OPT were re-contoured, to account for inter-fraction movement. The plan was adapted if tumor or OARs doses did not meet the constraints, as we prioritized OAR protection, even at the expense of PTV coverage. During radiation, the tumor was monitored by MRI, and treatment was automatically halted if the target (i.e., GTV) moved out of the boundary range by more than 5%. Breathing instructions were given to the patients during simulation and radiation sessions in order to minimize intra-abdominal organ movements and reduce treatment time.

All patients received oral Ondansetron prior to each radiation session to minimize possible nausea.

### 2.3 Assessment

Pre-treatment patient data included demographics, prior treatments, symptoms, and baseline tumor measurements to allow post-treatment calculation of Response Evaluation Criteria in Solid Tumors (RECIST) criteria (19) or PET-CT Response Criteria in Solid Tumors (PERCIST) (20). RECIST/PERCIST were used to determine local response alone, independently from disease status in distant sites (which was evaluated separately). Post-treatment assessment of local response was based on matching imaging modalities to minimize errors (i.e., MRI vs. MRI, PET-CT vs. PET-CT, and CT vs. CT).

Throughout and after the RT period, the treating radiation oncologist monitored patients’ acute and late (defined as occurring within or after 6 months of therapy completion, respectively) side effects such as gastrointestinal (abdominal pain, nausea, vomiting, diarrhea, constipation, and gastric outlet obstruction) and general side effects (fatigue, loss of appetite, weight loss, and anemia). Additionally, CA 19-9 levels were tracked at baseline and in the following months, and re-induction of chemotherapy was reported.

### 2.4 Statistical analysis

Associations between patient characteristics and side effects were evaluated by Mann–Whitney test, Spearman correlation, and chi-square test, as appropriate.

A linear mixed model for repeated measure analysis was used to evaluate individual CA 19-9 levels throughout the study follow-up among each group. To avoid multicollinearity, we verified that there are no correlations between independent variables that were included into the model. Disease-free period and overall survival were analyzed using Kaplan–Meier test. The level of significance used for all analyses was two-tailed and set at *p*<0.05. The SPSS statistical package (Version 28, SSPS Inc., Chicago, IL) was used for all statistical analyses.

## 3 Results

### 3.1 Patient characteristics

A total of 54 PC patients were treated with radiation using MR-LINAC with median age of 70.4 (45.2–85.9) years. A total of 48 patients (88.9%) had at least 60 days of follow-up at the time of evaluation. All but one patient (98.1%) had a biopsy-proven diagnosis of PC; most (96.2%) patients had pancreatic adenocarcinoma, one patient had adeno-squamous carcinoma, and one patient had cholangiocarcinoma. A total of 40 patients (74.0%) had primary inoperable PC (inoperable group), and 14 patients (25.9%) had local recurrence post Whipple procedure (operated group). One patient had undergone a preventive Whipple procedure 14 years prior to cancer diagnosis and therefore was regarded as inoperable PC. Twenty-one patients (38.9%) had metastatic disease at the time of radiotherapy.

Prior to SMART, 47 patients (87.0%) received chemotherapy, mainly using 5FU+Oxaliplatin-based regimen (n=39, 72.2%), and 14 patients (25.9%) received multiple sequential chemotherapy regimens following SMART. Six patients (11.1%), all previously operated, received RT to the pancreatic region prior to SMART, mostly chemoradiation (n=5, 9.25%) using either Capecitabine or Gemcitabine.

Apart from CA 19-9 serum levels, which were lower among operated patients compared to the inoperable group both at time of diagnosis (n=14, mean of 401, *p*=0.018 and n=38, mean of 1,877, respectively) and up to 6 weeks prior to RT (n=11, mean of 105, *p*=0.015 and n=30, mean of 978, respectively), there was no significant difference in demographics and baseline parameters between operated and inoperable groups. See **Table 1**.

**Table 1:** Patient, tumor, and prior therapy characteristics.

	Primary Inoperable Pancreatic Cancer (n = 40)	Local Recurrence Post Whipple Procedure (n = 14)	Total (n = 54)
**Median Age (range)**	70.3 (46.8-85.7)	64.6 (43.8-78.3)	69 (43.8-85.7)
**Sex**, n (%) Men Women	26 (65) 14 (35)	10 (71.4) 4 (28.6)	36 (66.7) 18 (33.3)
**Comorbidities**, n (%) IHD DM	6 (15) 16 (40)	3 (21.4) 6 (42.9)	9 (16.7) 22 (40.7)
**BRCA Status**, n (%) BRCA 1/2 Wild Type Unknown	0 (0) 20 (50) 20 (50)	0 (0) 4 (28.6) 10 (71.4)	0 (0) 24 (44.4) 30 (55.6)
**Smoking Status**, n (%) Currently Per History Non-Smoker Unknown	6 (15.8) 6 (15.8) 26 (68.4) 2 (5)	3 (23.1) 1 (7.7) 9 (69.2) 1 (7.7)	9 (17.6) 7 (13.7) 35 (68.6) 3 (5.6)
**Prior chemotherapy**, n (%) 1 protocol 2 protocols Oxaliplatin+5FU based Gemcitabine alone Gemcitabin based	34 (85) 6 (15) 32 (80) 1 (2.5) 8 (20)	13 (92.9) 8 (57.1) 7 (50) 4 (28.5) 4 (28.5)	47 (87) 14 (25.9) 39 (72.2) 5 (9.2) 12 (22.2)

IHD, Ischemic Heart Disease. DM, Diabetes Mellitus. RT, Radiotherapy. CRT, Chemoradiotherapy. C/G, Capecitabine / Gemcitabine.

### 3.2 Treatment characteristics

A total of 53 patients (98.1%) completed SMART to a prescription of 50 Gy in five fractions. On-table adaptive replanning was performed for 100.0% of all (269) fractions; one patient received four fractions due to intolerance for prolonged immobility necessary for accurate radiation delivery. After SMART, 32 patients (69.6%) received additional chemotherapy (26 inoperable patients and 6 recurrence patients).

### 3.3 Toxicity

None of the patients reported grade ≥2 acute GI toxicity or were hospitalized due to treatment-related side effects.

Prior to SMART, 40 patients (80.0%) reported disease-related symptoms, including abdominal pain (42.6%, n=23), back pain (14.8%, n=8), loss of appetite (27.7%, n=15), weight loss (42.5%, n=23), and fatigue (29.6%, n=16). Two inoperable patients (3.7%) had gastric outlet obstruction prior to SMART, which was resolved during treatments.

Apart from diarrhea at 1–3 months post-SMART and general fatigue, which were reported more frequently by previously resected patients in comparison to the inoperable group (30.0% vs. 10.0%, *p*=0.03 and 38.5% vs. 10.3%, *p*=0.033, respectively), no significant differences were found between the groups with respect to the remaining evaluated symptoms. All previously irradiated patients reported only low-grade toxicities during RT (100.0%, n=5, *p*=0.009).

### 3.4 Response and outcomes

A total of 48 patients (88.9%) were available for evaluation; one patient died due to his disease within 10 days following RT, and five patients were lost to follow-up. Median follow-ups from diagnosis were 22.3 and 8.9 months from SMART. As expected, the previously operated group had longer mean follow-up time from diagnosis compared to inoperable patients (40.3 and 20.3 months, respectively, *p*=0.001), and patients who received prior RT to the pancreas had an additional 8 months of follow-up time from SMART.

The first response and disease status were evaluated after 3.4 months in average. Complete local control was achieved by 21.7% (n=10) of patients for a median of 9 months (2.8–28.8 months); three patients had later local progression. Nine patients (19.6%) achieved partial response, and 21 patients (45.7%) had stable disease at the time of evaluation for a total of 87.0% local control. There was no significant difference between groups in local control, evidence of distant disease (new or known), and regional failure/relapse. None of the patients who underwent prior RT achieved CR; however, they still responded to treatment: one patient achieved partial local response (PR), and three patients had stable local disease (SD). The remaining two patients who had previously undergone RT had either died prior to post-SMART imaging evaluation or had locally progressive disease.

Five patients received concomitant radiation to liver metastases. Two patients were treated using a regular linear accelerator and had multiple new liver lesions upon radiological evaluation post-treatments. The remaining three patients were treated using SMART (45–50 Gy in five fractions); two patients had complete metabolic response in irradiated lesions, while the third patient had disease progression.

Mean CA 19-9 serum levels were consistently lower among previously resected patients and were significantly lower in 50.0% of evaluated time frames. See **Table 2** describing treatment outcomes.

**Table 2:** Outcomes Post SMART Treatment.

	Primary Inoperable Pancreatic Cancer	Local Recurrence Post Whipple Procedure	Total	P-Value
Local Response*, n (%) Complete Response Local Recurrence Partial Response Stable Disease Local Progression	8 (22.9) 3 (37.5) 8 (22.9) 14 (40.0) 5 (14.3)	2 (18.2) 0 (0.0) 1 (9.1) 7 (63.6) 1 (9.1)	10 (21.7) 3 (30.0) 9 (19.6) 21 (45.7) 6 (13.0)	NS NS NS NS NS
Distant Disease**, n (%) Prior to SMART Post SMART **Regional Failure/Relapse**	18 (45.0) 19 (54.3) 7 (19.4)	3 (21.4) 5 (45.5) 1 (9.1)	21 (38.9) 24 (52.2) 8 (17.0)	NS NS NS
**Chemotherapy Re-induction, n (%)**	26 (72.2)	6 (60.0)	32 (69.6)	NS
**Overall Survival (OS, in months)** Mean OS 6-months OS from SMART completion 12-months OS from SMART completion 18-months OS from SMART completion	15.1 75.0% 50.0% 41.6%	11.8 83.3% 58.3% 41.6%	14.43 75.0% 52.1% 41.6%	NS NS NS NS

* Local Response was determined based on RECIST & PERCIST criteria

** Distant disease existence was evaluated based on imaging. Post SMART metastatic disease was determined based on the same imaging study used to evaluate local response to treatment.

Dx, Diagnosis. SMART, Stereotactic MR-guided Adaptive Radiotherapy. NS, Non-significant, P>0.05.

**Figure 2:**
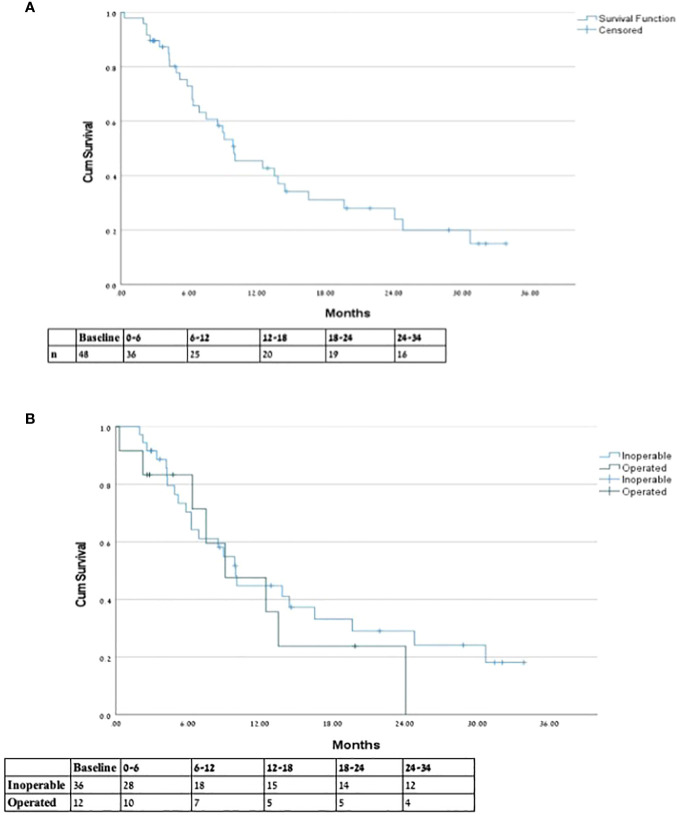
Overall survival from end of SMART. * Overall survival from end of SMART for **(A)** entire cohort and **(B)** inoperable vs. previously operated patients.

OS of 6 and 12 months (from end of SMART) was 75.0% and 52.1%, respectively. Operated patients had an understandably longer mean survival from diagnosis compared to inoperable patients (49.85 and 24 months, respectively); however, there was no significant difference between groups in 6-, 12-, and 18-month OS from end of SMART. See **Figure 2** comparing OS between groups. Non-metastatic patients had a 6, 12, and 18 months OS of 55%, 44%, and 28%, respectively. The type of local response to treatment had no significant impact on survival (*p*=0.935). Patients with previous RT (five patients with more than 60 days follow-up) had 6 months OS of 80%, and one patient remained alive after 12 months.

## 4 Discussion

In this study, we report our experience on treating pancreatic cancer, either locally advanced or recurrent, with high-dose adaptive MR-guided radiation therapy. We found that SMART is safe, with minimal treatment-related toxicity, even in previously irradiated patients, and that both operated and inoperable patients can achieve local response, with over 20% rate of complete local response to treatment.

By including patients with metastatic disease, local recurrence post-Whipple, and previously irradiated patients, our study population differed greatly from that of previous studies. To the best of our knowledge, published SMART studies have focused mainly on treating inoperable locally advanced PC in patients without prior RT (15, 21), frequently excluding metastatic disease as well (16, 22, 23), unless the study purpose was to examine re-irradiation specifically (18).

Similar to previously published research with MR-guided SBRT, our prescribed dose to inoperable PC was 50 Gy in five fractions, with special attention to daily on-table adaptation, and 100% of the delivered fractions was adapted. Treatment plan adaptation poses an important aspect in our treatment plan, ensuring that OAR tolerance doses are not exceeded, even at the expense of PTV coverage. Indeed, toxicity was minimal during and after treatment, even in previously resected or irradiated patients. None of the patients experienced grade ≥ 2 toxicities. This is in concordance with other reports with very low toxicity rates. **Table 3** contains a summary of selected studies of SMART in PC. For example, in their research, Henke et al. (15), Rudra et al. (16), Hassanzadeh et al. (22), and Chuong et al. (23, 24) treated patients with a comparable prescription dose (BED_10_ over 70 Gy) using SMART and reported aligning results—0.0%, 0.0%, 4.6%, 2.9%, and 8.8% grade ≥ 3 acute toxicities, respectively. In the study by Hassanzadeh et al., some patients received RT using MRIdian Cobalt-60 system, with 4.6% reported associated grade 3 toxicity for all patients with no further information (22). As treatment volumes at the phase II study by Parikh et al. were at the discretion of the treating physician, some patients were treated to adjacent anatomic regions considered to be at high risk for micro-metastatic disease (24). This may contribute to the reported higher rate of grade 3 toxicity, 8.8%. Conventional fractionation (BED_10_ 55.5 Gy) resulted in 15% grade 3 or higher in the study by Rudra et al. (16). These are highly encouraging results, as PC is known for posing a challenge for the treating radiation oncologist due to its proximity to delicate GI structures (25). It should be noted that in our treatment protocol, all patients were prescribed Ondansetron to prevent radiation-related nausea, which might explain the low rate of nausea complaints.

**Table 3:** Summary of selected studies of SMART in pancreatic cancer.

Study	Patient number	Total dose and fractions	BED_10_	Median follow-up (months)	LC	OS	Acute grade 3+ toxicity (%)
*Henke et al. (2018)* (15)	5	50 Gy × 5	100	15	6-months 89.1%	1-year 75.0%	0.0%
*Rudra et al. (2019)* (16)	24+	40–52 Gy × 5	72–106.1	17	2-year 77.0%	2-year 49.0%	0.0%
*Placidi et al. (2020)* (21)	8	30–40 Gy × 5	48–72	13	25.0%	87.5% at last F/U	0.0%
*Hassanzadeh et al. (2021)* (22)	44	50 Gy × 5	100	16 (from dx)	1-year 84.3%	1-year 68.2%	4.6%
*Chuong et al. (2020)* (23)	35	40–50 Gy × 5	100	10.3	1-year 87.8%	1-year 58.9%	2.9%
*Chuong et al. (2021)* (17)	148	40–50 Gy × 5	100	16 (from dx)	1-year 94.6%	1-year 82.0%	4.1%
*Chuong et al. (re-irradiation, 2022)* (18)	11^++^**	40 Gy × 6	44.7	14	1-year 88.9%	1-year 70.0%	0.0%
*Parikh et al.* *(2023)* (24)	136	50 Gy × 5	100	8.8	1-year 82.9%	1-year 65.0%	8.8%
*Current study*	54	50 Gy × 5	100	8.9	87.0%*	1-year 52.08%	0.0%

BED, biologically effective dose; LC, local control; OS, overall survival.

^+^ Including nine patients with hypofractionated protocol, with median BED_10_ of 82.7.

^++^ Including four patients with hypofractionated dose schedule.

* Local control for this study was calculated as the percentage of patients who had either complete local response, partial response, or stable disease at time of first radiological evaluation post-SMART.

** Re-irradiation for multiple malignancies. Out of 11 patients, only 3 patients had PC.

The main endpoints of this study were local control and outcomes. We report that 87.0% of treated patients achieved local control at the time of evaluation, whether in the form of complete local response (CR, 21.7%), partial response (PR, 19.6%), or stable disease (SD, 45.7%), while only 13.0% had local progressive disease (LPD). Due to the frequent use of PET-CT as a physiological imaging modality, we were able to evaluate disease metabolic status using PERCIST criteria (20). Although this allowed for a most accurate evaluation of therapeutic effects, local metabolic response in the form of CR/PR/SD/LPD, and whole-body disease status (26), we find ourselves unable to compare these results to previously published studies, in which “local control” was the main endpoint evaluated by CT scans.

However, as opposed to previously published articles, our research reports 1-year OS of 58.3%, which is considerably lower in comparison to others (15, 17, 18, 22, 23). This could be attributed to the difference in patient selection, as our study population includes metastatic disease or previous local surgical/radiation treatments to the pancreas, suggesting a more advanced disease.

The singularity of this study lies in the comparison of outcomes between previously operated and inoperable patients. We aimed to evaluate differences in outcome and toxicity of SMART between these two groups to better understand the role of patient selection based on disease and treatment history. We found that there were no significant differences in demographics and response to treatment between the groups, even among patients who have previously undergone RT to the pancreas, suggesting that SMART can potentially benefit patients regardless of previous treatment attempts.

In our study, we found two major areas distinguishing the two groups, the first being CA 19-9 serum levels, which were consistently lower among operated patients in most evaluated time frames, suggesting that inoperable patients had a more advanced disease or that they had a disease less susceptible to treatments. Additionally, we noticed that previously operated patients tended to experience more short-term (although low-grade) toxicities, such as fatigue and diarrhea. While diarrhea could be sporadic or a result of pancreatic endocrine insufficiency, in their review, Chang et al. (27) show that fatigue is more common in previously operated patients in comparison to inoperable patients (73% and 53%, respectively). Thus, it is possible that these findings are essentially disease-related symptoms as opposed to treatment-related toxicities. For example, disease-related fatigue could be supported by longer follow-up time from diagnosis among the operated group, expressing that these patients have been coping with PC diagnosis and systemic treatment for longer time periods. As this is the first research to evaluate the difference in outcomes and toxicities after SMART between operated and inoperable patients, unfortunately and to the best of our knowledge, there is no available literature to compare our results to. Further research is needed to evaluate the difference in SMART-related toxicities in inoperable and previously operated patients regardless of RT-related symptoms.

This study has some limitations. This is a single-center study; however, it is a relatively large series compared to other single-center studies. This is a retrospective study, subject to under-reporting toxicities, although toxicities were documented prospectively. Additionally, our results could benefit from a more extended follow-up to better understand late toxicity and long-term clinical outcomes. Finally, whereas local response was an endpoint for other studies, in this study, physiological imaging modalities (MRI and PET-CT) were used to evaluate local response. This posed a challenge in terms of comparing results, yet we suggest that this also allowed for a more accurate evaluation of local response, with a potential for standardization thanks to the use of RECIST and PERCIST criteria.

## 5 Conclusion

SMART is a safe local treatment modality for pancreatic cancer, with minimal treatment-related toxicity, even in previously resected or irradiated patients. Local control with complete response was achieved by 21.7% of patients, regardless of previous surgical history. Further studies are needed to evaluate long-term outcome and late toxicity and to identify significant factors for patient selection.

## Data availability statement

The raw data supporting the conclusions of this article will be made available by the authors, without undue reservation.

## Ethics statement

The studies involving humans were approved by Assuta Medical Center Helsinki Committee. The studies were conducted in accordance with the local legislation and institutional requirements. Written informed consent for participation was not required from the participants or the participants’ legal guardians/next of kin in accordance with the national legislation and institutional requirements.

## Author contributions

GA: Writing – original draft, Writing – review & editing. RP: Writing – review & editing. SZ: Writing – review & editing. VG: Writing – review & editing. YLi: Writing – review & editing. EC: Writing – review & editing. DLe: Writing – review & editing. SA: Writing – review & editing. OA: Writing – review & editing. DE: Writing – review & editing. QT: Writing – review & editing. KH: Formal analysis, Writing – review & editing. DLi: Writing – review & editing. TG: Writing – review & editing. IB-A: Writing – review & editing. YRL: Writing – review & editing. MB-D: Conceptualization, Methodology, Project administration, Supervision, Validation, Writing – review & editing.

## Footnotes

1 V33 = volume of tissue or organ that receives a radiation dose of 33 Gy or higher.

2 V25 = volume of tissue or organ that receives a radiation dose of 25 Gy or higher.
